# Micro-costing the provision of emotional support and information in UK eye clinics

**DOI:** 10.1186/1472-6963-13-482

**Published:** 2013-11-19

**Authors:** Hanna Gillespie-Gallery, Ahalya Subramanian, Miriam L Conway

**Affiliations:** 1 Division of Optometry and Visual Science, City University London, Northampton Square, London EC1V 0HB, UK

**Keywords:** Cost analysis, Micro-costing, Allied health personnel, Emotional support

## Abstract

**Background:**

Sight loss has wide ranging implications for an individual in terms of education, employment, mobility and mental health. Therefore there is a need for information and support to be provided in eye clinics at the point of diagnosis of sight threatening conditions, but these aspects of care are often missing from clinics. To meet these needs, some clinics employ an Eye Clinic Liaison Officer (ECLO) but the position has yet to be widely implemented. The aims of this study were:

(1) To evaluate the forms of advice and emotional support in eye clinics provided by ECLOs.

(2) To determine the cost of the ECLO service per patient.

**Methods:**

Micro-costing was carried out using interviews, a survey and administrative data. The survey was completed by 18 of the 49 accredited ECLOs in the UK (37%) and provided information on the activities performed by ECLOs, numbers of patients seen per day, training costs incurred and the salary of the ECLOs.

**Results:**

ECLOs provided information about the services in eye clinics and the community, referral to social services, emotional support to patients and also other advice. The cost of an ECLO per patient per contact was £17.94 based on an average annual ECLO salary of £23,349.60 per year, reviewing on average 9.1 patients per day, in a 42 week year.

**Conclusions:**

This study provides the first costing of support services in hospital eye clinics, providing a range of estimates to suit the circumstances of different clinics. The information can be used by local decision makers to estimate the cost of implementing an ECLO service.

## Background

There is a growing recognition that better support mechanisms are needed for patients in eye clinics, particularly at the time of sight loss. Previous research conducted in the UK suggests that eye clinic patients with sight loss do not always receive emotional support [1,2] or information about services and low vision aids available to them [3,4]. Eye care professionals suggest there is an under provision of these services, and are often unsure who provides information and/or support [5]. The role of the Eye Clinic Liaison Officer (ECLO) was established to address these problems. ECLOs work in eye clinics providing information and emotional support (attentive listening and constructive suggestions) to patients of all ages and their families. They provide a single point of contact for information about registration and certification as sight impaired and severely sight impaired (partially sighted/blind), eye conditions, information about education, employment and other social services and then can refer patients to statutory and voluntary organisations [6]. In an eye clinic a clinician may refer a patient to an ECLO or the patient may approach an ECLO independently to obtain information and/or support.

Despite the growing awareness about the need for timely support, the ECLO service has not been widely implemented. Other countries have implemented a similar support/co-ordinator role such as the USA, Australia, Sweden and Poland, but in some countries the role is only available within visual impairment organisations in the community rather than in eye clinics [7]. The utility of having an ECLO within an eye clinic means that patients have access to emotional support at the point of diagnosis and then can be advised how to contact social services and other organisations.

One of the reasons why the ECLO service has is not been widely implemented may be due to financial concerns and a lack of cost estimates available in the literature. In a recent report of the key cost drivers for low vision services in the UK, concern was raised over the sustainability of the ECLO role due to cost factors, despite the general feeling that these individuals played a valuable role in low vision services [8]. To provide an estimate of costs, we used micro-costing, a method often used to estimate the costs of new services [9]. A recent study used micro-costing to evaluate the costs for low vision appointments at an optometry led hospital service found that a low vision appointment costs £165 per patient in the financial year 2007–2008 [10]. In another study, the cost of a hospital glaucoma clinic was estimated at £63.91 per patient [11].

The aims of this study were to determine (1) whether ECLOs are providing the services previously mentioned as missing in eye clinics, and (2) the cost of an ECLO per patient per contact and whether sufficient facilities have been provided for ECLOs. Cost per patient per contact is the cost of one consultation with an ECLO.

## Methods

An invitation was sent to ECLOs known to the researchers to take part in semi-structured interviews to inform items for use in the questionnaire (for questions, see Additional file 1, questions). Four ECLOs agreed to take part. The online questionnaire was piloted using one ECLO and one individual who had previously worked as an ECLO and minor changes were made based on their comments. The authors contacted some ECLOs directly and the Royal National Institute of Blind People (RNIB) sent out an invitation by email to take part to ECLOs known to them. All responses were anonymous and questions were optional. The study was approved by City University London’s Research and Ethical Committee and the study adhered to the tenets of the declaration of Helsinki.

### Determining the services provided by ECLOs

ECLOs were asked in the survey which information and support activities they provided to patients, and selected a yes/no answer. The activities are provided in Table 1.

**Table 1:** Responses to the question “Which of these general activities do you undertake with patients/clients?”

**Service**	**ECLOs providing service (%)**	**Total number of respondents**
Information about the low vision clinic	100.0	18
Signposting to other sources of help (including charities and advice about housing, education, benefits and employment)	100.0	18
Explanation of the process of registration	100.0	16
Referring to social services	100.0	18
Explanation of the cause of the patient/client’s vision loss and prognosis	94.4	18
Explanation about the use of non-optical aids e.g. Lighting	88.9	18
Explanation about the use of Low Vision Aids	88.2	17
Training in the use of Low Vision Aids	35.7	14
Emotional Support (excluding counselling)	100.0	18
Family Support	94.4	18

### Estimating the costs of ECLOs per patient and facilities for ECLOs

Table 2 outlines the costs used to calculate the cost of an ECLO per patient contact. Information to estimate costs was obtained from three sources. The survey determined how many patients ECLOs saw per day, training costs incurred and the salary of the ECLOs. The survey also asked about the facilities provided for ECLOs. Unit Costs for Social and Health Care [12] contributed to the calculation of the patients seen per year (1911), employer national insurance and superannuation/pension contributions (£5,527.70), overheads such as telephone, heating and stationary (£3,130.00) and capital overheads such as building and fittings costs (£2,283.00). An unpublished ECLO business case proposal estimated the set up costs for an ECLO (£3,110.00).

**Table 2:** Figures used to estimate the average cost of an ECLO per patient contact

		**Source and notes**
**Average patients per day**	9.1	Survey responses n= 15, SD=8.2
**Patients per year**	1911	Assuming 42 working weeks per year, based on data from Unit Costs for Social and Health Care [12]. Includes 29 days annual leave and 8 statutory leave days. Assumes 6 study/training days and 12 days of sickness leave.
**Average annual salary**	£23,349.60	Survey responses n=15, SD=£3,818.36
**Salary on-costs (Includes Employers NI costs + 14% of employers salary for superannuation/pension costs)**	£5,527.70	Based on data from Unit Costs for Social and Health Care [12].
**Average training cost**	£760.00	Survey responses n=10, SD=£39.49
**Set up cost**	£3,110.00	Data from an unpublished ECLO business case proposal.
**Overheads**	£3,130.00	Based on data from Unit Costs for Social and Health Care [12]. Capital Overheads include the cost of office space and land costs.
**Capital Overheads**	£2,283.00	
**Full economic cost to employ an ECLO (year 1)**	£38,161.42	Includes training and set-up costs.
**Full economic cost to employ an ECLO (year 2)**	£34,290.30	Assumes no training costs or set-up costs.
**Total cost per contact (Year 1)**	£19.97	Includes training and set-up costs.
**Total cost per contact (Year 2)**	£17.94	Assumes no training costs or set-up costs.

The full economic cost for year one, when the service is initially implemented, is calculated by summing all costs, and the costs for year two are calculated in the same way but removing the set up and training costs. The cost per patient per contact was calculated by dividing the full economic costs by the estimated number of patients seen per year.

The number of patients an ECLO see per day may vary from clinic to clinic [13]. Therefore a sensitivity analysis was conducted. Using the same method as in Table 1, the cost of an ECLO per patient contact was calculated for 5, 10 and 15 patients seen per day (Table 3).

**Table 3:** Costs per contact (without year 1 implementation costs) with different numbers of patients per day

**Number of patients per day**	**Cost per patient**
5	£32.66
9.1 (average)	£17.94
10	£16.33
15	£10.89

The cost of an ECLO intervention over a person’s lifetime was calculated for five age points assuming a person consulted an ECLO once per year, once every two years and once every five years. The following age points were used as data on life expectancy and percentage of the population that are male and female were available for these ages: 8, 40, 70, 80 and 84. The values for contact once per year were calculated as follows:

[Proportion female x average female lifetime remaining × cost per contact] + [proportion male × average lifetime remaining x cost per contact] [14,15].

The average cost of an ECLO per lifetime for a clinic as a whole was calculated by weighing the proportions of people registered as severely sight impaired at each age group [16].

## Results

### Description of ECLOs

The study took place in June 2010. At this time, the RNIB estimated that there were 49 individuals who had completed the City University London accredited RNIB ECLO training course and were working as ECLOs. 18 out of the 23 ECLOs who participated in the survey were accredited and worked more than one full day per week (37% of all accredited ECLOs) and only the results of accredited ECLOs are described. Most ECLOs had the job title of Eye Care/Clinic Liaison Officer (n=13), other titles included Low Vision Co-ordinator/Support Worker (n=3), Information Officer (n=1) and Independent Living Advisor (n=1). Funding for the ECLO position was provided by local authorities (n=5), NHS (n=5), jointly by the local authority and NHS (n=2), local charities (n=2) and national charities (n=4).

### Role of ECLOs

The information and support activities provided by ECLOs are shown in Table 1. All questions were optional, and the ‘Total number of respondents’ column provides information on how many people responded to each part of the question. Most services are provided by 90% or more ECLOs. Slightly fewer ECLOs provided explanations about low vision aids (LVAs; 15 out of 17, 88.2%) and non optical aids, such as lighting (16 out of 18, 88.9%) and only 5 out of 14 ECLOs (35.7%) provided training in the use of LVAs. Training in LVAs is likely to be outside the remit of most ECLOs.

### Cost of providing a full time ECLO in eye clinics

Table 2 shows that the full economic cost of an ECLO per year is £34,290.30. The cost per patient per contact with the ECLO was £17.94 for seeing an average of 9.1 patients per day. These figures do not include initial set-up costs or training costs. £17.94 is carried forward for subsequent calculations.

### Sensitivity analysis

Table 3 shows that the cost of an ECLO per patient contact varies from £10.89 if 15 patients are seen per day, to £32.66 if 5 patients are seen per day.

### Cost of providing support per lifetime per patient

Table 4 shows the cost of an ECLO intervention over a person’s lifetime for ages 8–84 years. Costs vary from £24.60 for an 84 year old who visits and ECLO once every 5 years to £1,300.92 for an 8 year old who visits an ECLO once per year for their remaining life.

**Table 4:** The lifetime cost of an ECLO’s intervention for various ages and frequencies of contact

**Age**	**8**	**40**	**70**	**80**	**84**
Once per year	£1,300.92	£742.56	£275.75	£159.03	£122.99
Once every 2 years	£650.46	£371.28	£137.88	£79.51	£61.49
Once every 5 years	£260.18	£148.51	£55.15	£31.81	£24.60

Using the proportions of people registered as severely sight impaired at each age group [15] we estimate the average lifetime cost of an ECLO for an eye clinic is £247.76, assuming an ECLO is seen once per year.

### Facilities provided to ECLOs

ECLOs were asked what facilities were provided for their role in the hospital. 72.2% had a private room, 66.7% had a telephone and 44.4% had a computer (n=18). When asked if they were satisfied with the facilities provided, only 44.4% answered yes. ECLOs who provided additional comments about facilities often mentioned that a lack of space or sharing the space with other staff made it difficult to discuss patient’s private situations and to provide emotional support.

## Discussion

### Services provided by ECLOs

ECLOs provide information about low vision clinics, social services and signposting to other agencies. Emotional support to patients and family members is widely provided. Just over 10% do not provide information about low vision aids, and most do not provide training in their use, however, this is provided in the community [5] and may be outside the remit of many ECLOs.

### The cost of an ECLO

After the first year of set-up costs, the full economic cost of employing a full time ECLO is £34,290 per annum, and £17.94 per patient per contact. This increases the estimated cost of a hospital based low vision service [10] by 10.8%. We do not include costs to patients, as the patient would see the ECLO in addition to clinicians, but this has previously been estimated as £6.15 to attend a hospital clinic [11].

The sensitivity analysis showed that cost per patient could range from £32.66 to £10.89 if 5 or 15 patients were seen per day respectively. The variations in cost highlight the importance of effectively utilising the service once implemented. However, some variation is inevitable due to the geographical distribution of the elderly population, for example, there are greater proportions of elderly people in coastal areas in the UK due to migration [13]. Although there are advantages to increasing the number of patients seen per day by an ECLO in order to decrease the cost per patient, there is a limit. Previous research has indicated that on average, face-to-face contact takes 30 minutes and subsequent administration 15 minutes per patient [17], resulting in almost 7 hours of work per day for the average of 9.1 patients being seen. The amount of time required per patient will vary based on the individual’s requirements and situation, such as prior knowledge of their eye condition, changes in health or circumstances and the availability of emotional support within their social network. Ultimately, an eye clinic will need to take these factors into account to determine how well utilised the service will be, without exceeding capacity.

The average lifetime cost of an ECLO per patient ranged from £260.18 for someone first seeing an ECLO at age 8, and £24.60 for someone first meeting an ECLO at age 84, assuming an ECLO is met once every 5 years. Although meetings with ECLOs are not meant to be provided regularly to patients, as they refer patients on to service providers, the changing social needs of patients as a result of progressing visual impairment and/or the changing demands put on an individual at each age may require more regular meetings for advice about education, employment, childcare, welfare rights, benefits and other developing conditions and this must definitely be borne in mind when looking at the cost of the service. Culham et al. [13] suggest that most people needing assistance with visual impairment would need a minimum of 3 appointments at the clinic over their lifetime.

Although ECLOs are providing the services reported as previously missing in eye clinics [5], earlier research has not found that ECLOs provided an improvement in measures such as quality of life or adaptation to vision loss [17,18], thus a cost-benefit analysis could not be performed. Interventions resulting in measurable improvements tend to be intensive in duration i.e. [19] or involve training in low vision aids i.e. [20], both of which are outside the scope of the ECLO role. The strength of the ECLO role is to provide immediate emotional support and information and referrals to other services, rather than providing an intervention itself.

### Facilities available for ECLOs

ECLOs were not always provided with facilities to carry out their role effectively. Computers and telephones, useful for making referrals and obtaining information for patients, were only available to 44.4% and 66.7% respectively. Although a private room was the resource most ECLOs had access to (72.2%), those without this commented it caused many problems as there was no place to discuss a patient’s situation and feelings. In addition most ECLOs were unsatisfied with the resources available to carry out their role.

### Limitations

There are certain limitations to our study which may have resulted in an underestimation of costs. The cost per contact was based on self-report of the average number of patients (9.1), and a smaller sample diary study of ECLOs reported an average number of 6 patients per day [21]. However, the sensitivity analysis provides costs for different number of patients per day, so values suitable for a particular situation can be chosen. The costs do not include any additional training for the ECLO such as continuing education and training costs. It was difficult to find estimates of ongoing training costs, hence these were not included. For estimation of the average lifetime costs, we assumed that life expectancy is not reduced by visual impairment. Although this is unlikely due to increasing evidence of the link between visual impairment with falls [22,23] and car accidents [24]. The calculations used the midpoint of age groups in some parts of the calculations (e.g. life expectancy for 70 year olds) and broader age groups for other parts of the calculation (proportion registered severely sight impaired) because the data was collected from a range of sources. Despite this we feel that our study provided a reasonable estimate of what it would cost to provide emotional and support services for people with visual impairment.

Our response rate was 37% of all accredited ECLOs. One potential reason for the low response rate could be due to the sensitive nature of some questions, in particular those regarding salary. In addition, only 44.4% of ECLOs reported they had access to a computer. Thus, many may have completed the survey at home/elsewhere, and perhaps some ECLOs did not want to answer the questionnaire outside their working hours.

## Conclusions

In conclusion, ECLOs are providing previously missing information and support to patients and their families in eye clinics. However, ECLOs may not always be able to provide these services effectively due to a lack of facilities. Implementing an ECLO service may increase the cost of a low vision clinic by approximately 10.8%, but Hodge et al. [25] suggest that the provision of emotional support and counselling to visually impaired individuals results in better uptake of rehabilitation services, less need for medication, less psychosomatic illness and less need for primary care services. Whether the service is cost effective should be evaluated in future when the service is better established and standardised.

## Abbreviations

ECLO: Eye Clinic Liaison Officer; LVA: Low Vision Aid; RNIB: Royal National Institute of Blind People.

## Competing interests

The authors declare that they have no competing interests.

## Authors’ contributions

AS and MC conceived of the study and HGG acquired the data. All authors contributed to the design of the study, analysis and interpretation of the data, the drafting and revision of the manuscript and final approval of the manuscript.

## Pre-publication history

The pre-publication history for this paper can be accessed here:

http://www.biomedcentral.com/1472-6963/13/482/prepub

## Supplementary Material

Additional file 1 Questions.pdf contains a list of questions used in the survey to ECLOs. Click here for file
