# Supplementary material for: Micro-costing the provision of emotional support and information in UK eye clinics
Source: BMC Health Serv Res. 2013 Nov 19;13:482. doi: 10.1186/1472-6963-13-482 (PMC3842648; doi:10.1186/1472-6963-13-482)
Supplement: Additional file 1 — Questions.pdf contains a list of questions used in the survey to ECLOs. [file 1472-6963-13-482-S1.docx]

**List of questions used in the survey to ECLOs**

Below is a list of questions used in the survey to ECLOs. The questions were part of a larger survey however only shown are questions relevant to the paper, “Micro-costing the provision of emotional support and information in UK eye clinics”.

**1. Which of the following options represent your terms of work (multiple options can be selected)?**

- Part time
- Full time
- Permanent
- Contract
- Job share
- Other (please specify) _________

**2. How many hours do you work as an ECLO per week?** _________

**3. What is your job title?**

- Eye Care Liaison Officer
- Point of Diagnosis Officer
- Other (please specify) _________

**4. Which of these general activities do you undertake with patients/clients?**

**9. Activities undertaken with patients/clients (1)**

|  | **Yes** | **No** |
| --- | --- | --- |
| Information about the low vision clinic | ⭘ | ⭘ |
| Signposting to other sources of help (including charities and advice about housing, education, benefits and employment) | ⭘ | ⭘ |
| Explanation of the process of registration | ⭘ | ⭘ |
| Referring to social services | ⭘ | ⭘ |
| Explanation of the cause of the patient/client’s vision loss and prognosis | ⭘ | ⭘ |
| Explanation about the use of non-optical aids e.g. Lighting | ⭘ | ⭘ |
| Explanation about the use of Low Vision Aids | ⭘ | ⭘ |
| Training in the use of Low Vision Aids | ⭘ | ⭘ |
| Emotional Support (excluding counselling) | ⭘ | ⭘ |
| Family Support | ⭘ | ⭘ |

**6. Did you complete any special training to become an ECLO?**

| Yes | ⭘ |
| --- | --- |
| No | ⭘ |

**7. If known, how much did the course cost?** _________

**8. What is your annual salary (pro rata)? Please specify a figure (before tax) or an NHS pay band and point.** _________

**9. Who funds your position?**

- Primary Care Trust (PCT)
- Local authority
- Action for Blind People (AFBP)
- Royal National Society of Blind People (RNIB)
- Other (please specify)

**10. Please specify the number of patients/clients seen on an average day.** _________

**11. What facilities are provided for you at the hospital?**

|  | **Yes** | **No** |
| --- | --- | --- |
| Private room/office | ⭘ | ⭘ |
| Computer | ⭘ | ⭘ |
| Telephone | ⭘ | ⭘ |
| Storage space | ⭘ | ⭘ |
| Others (please specify) | ⭘ | ⭘ |

**12. Are you satisfied with the facilities made available to you at the hospital(s) you work at?**

**7. W**

| Yes | ⭘ |
| --- | --- |
| No | ⭘ |
| Unsure | ⭘ |
| Additional comments |  |
